# Target-Response Associations Can Produce Response-Congruency Effects Without Task-Switching Costs

**DOI:** 10.3389/fpsyg.2019.00040

**Published:** 2019-02-11

**Authors:** Bingxin Li, Xiangqian Li, Xuhong Liu, Martin Lages, Gijsbert Stoet

**Affiliations:** ^1^School of Psychology, University of Glasgow, Glasgow, United Kingdom; ^2^Department of Psychology, School of Social Development and Public Policy, Fudan University, Shanghai, China; ^3^Department of Psychology, University of Essex, Colchester, United Kingdom

**Keywords:** task-switching, bivalent stimuli, target-response association, task-switching cost, congruency effect

## Abstract

In task-switching experiments with bivalent target stimuli, conflicts during response selection give rise to response-congruency effects. Typically, participants respond more slowly and make more errors in trials with incongruent targets that require different responses in the two tasks, compared to trials with congruent targets that are associated with the same response in both tasks. Here we investigate whether participants show response-congruency effects when task rules are not made explicit. In two experiments, we assigned task-irrelevant features to each bivalent target. When participants were instructed to apply the task rules, they showed significant task-switching costs as well as response-congruency effects. Importantly, when the same participants did not know the task rules and responded without applying the task rules, they showed response-congruency effects but no switch costs. The significant congruency effects suggest that associations between bivalent target features and responses can be formed passively, even when participants do not follow the task rules and use task-irrelevant target features to make a response.

## Introduction

In task-switching experiments, researchers typically distinguish not only between repeat and switch trials but also between trials with congruent and incongruent target stimuli (Wendt and Kiesel, 2008; Schneider, 2015, 2017). Consider, for example, a task-switching paradigm with randomly intermixed color and shape tasks. The color task requires categorizing a target as black or white. The shape task involves categorizing a target as a circle or a hexagon. White and circular targets are associated with a left response key and black and hexagonal targets are associated with a right response key. In this example, a white circle and a black hexagon are congruent targets that lead to the same response in the color and shape task. Incongruent targets, black circles and white hexagons, lead to different responses in each task. For example, a black circle is associated with the right key in the color task because the color is black; it is associated with the left key in the shape task because it has a circular shape. Participants typically have increased response times (RTs) and error rates (ERs) in trials with incongruent targets compared to congruent targets. These differences in RT and ER are known as response-congruency effects (Sudevan and Taylor, 1987).

It has been suggested that response-congruency effects can be the result of response selection in two different processing routes: a *non-mediated* route (Kiesel et al., 2007; Wendt and Kiesel, 2008; Waszak et al., 2013; Schneider and Logan, 2015) and a *mediated* route (Meiran and Kessler, 2008; Schneider and Logan, 2009, 2014; Reisenauer and Dreisbach, 2014; Schneider, 2015, 2017).

In the non-mediated route, target-response associations are retrieved directly from working memory (Kiesel et al., 2007; Wendt and Kiesel, 2008; Waszak et al., 2013; Schneider and Logan, 2015) bypassing any intermediate target-feature categorization. Response selection should be faster and ERs should be lower in trials with congruent targets because they are only associated with a single response. In contrast, incongruent targets are associated with different responses in two tasks, resulting in conflicting target-response associations. Participants need to take the task cue into account when resolving the conflict between two target-response associations. Deducing the correct response in this way results in increased RTs and ERs. In order to explain this deterioration in performance, the mediated route has been suggested.

The mediated route requires participants to categorize target features according to the task rules before they can select a response (Meiran and Kessler, 2008; Schneider and Logan, 2009, 2014; Reisenauer and Dreisbach, 2014; Schneider, 2015, 2017). The response-congruency effects emerge as a consequence of conflicting feature-response selection in incongruent trials.

It has been suggested that the mediated route can produce response-congruency effects independently of the non-mediated route. For example, researchers reported response-congruency effects even when target stimuli were not repeated and appeared only once in the entire experiment (Liefooghe et al., 2012; Schneider, 2015, 2017). By presenting each target only once the non-mediated route can be avoided because participants did not learn to associate targets with responses. The response-congruency effects were the result of rule-based feature categorization and conflicting feature-response selection in the mediated route (Liefooghe et al., 2012; Schneider, 2015, 2017).

**FIGURE 1:**
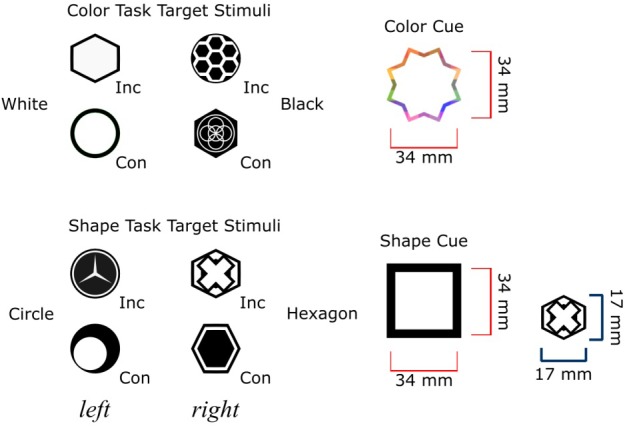
Illustration of the stimuli in Experiment 1. Four unique target stimuli were presented in the color task (top left), and four unique target stimuli were presented in the shape task (bottom left). Con = trial with congruent target; Inc = trial with incongruent target. Target stimuli based on white circles and black hexagons were congruent targets because both task-features (i.e., color and shape) lead to the same response in the two different tasks according to the task rules. Target stimuli based on black circles and white hexagons were the incongruent targets because each task-feature lead to different responses according to the task rules. Each target had at least one distinctive task-irrelevant feature.

However, if target and responses are repeated then feature-response associations can be formed in a passive or automatic learning process that does not require task rules (e.g., Hommel, 1998, 2004). In the present study, we focused on possible contributions from both task-relevant and task-irrelevant target features on response-congruency effects. In two experiments, each of the bivalent targets had two task-relevant features that afforded two tasks (color and shape task). The two task-relevant features were associated with the same response key in congruent trials and different response keys in incongruent trials (Figure 1). According to the mediated route, participants who apply the task rules and categorize the task-relevant target features should show clear response-congruency effects (Meiran and Kessler, 2008; Schneider and Logan, 2009, 2014; Reisenauer and Dreisbach, 2014; Schneider, 2015, 2017). In the present studies, each target was also given unique features that were irrelevant to the color and shape task. In the following we call these features unique task-irrelevant features.

The unique task-irrelevant features of each target can be associated with a single response. If participants learn to use these associations without applying task rules, then they should not experience a conflict during response selection, similar to studies with univalent stimuli (e.g., Dreisbach et al., 2006, 2007; Dreisbach and Haider, 2008). Thus, the non-mediated route of response selection should not result in congruency effects. However, our target stimuli also had bivalent or task-relevant features. If bivalent feature-response associations can be formed passively (e.g., Hommel, 1998, 2004), then this may lead to conflicts during response selection in trials with incongruent targets. Here, we sought to establish whether participants who do not apply task rules show such response-congruency effects.

In addition to response-congruency effects, we also monitored task-switching costs. According to the task reconfiguration and/or proactive interference account, switching between tasks should result in longer RTs and higher ERs (Kiesel et al., 2010; Vandierendonck et al., 2010), irrespective of the use of univalent targets (Dreisbach et al., 2006, 2007; Dreisbach and Haider, 2008) or bivalent targets (e.g., Forrest, 2012; Forrest et al., 2014). As a consequence, participants who apply task rules should show significant task-switching costs.

It has been suggested that participants who use associations between univalent targets and responses were able to eliminate task-switching costs (Dreisbach et al., 2006, 2007; Dreisbach and Haider, 2008), whereas associations between bivalent targets and responses showed residual task-switching costs (Forrest et al., 2014; Forrest, 2012; but see Li et al., in press). Forrest et al. (2014) argued that an associative learning network can generate task-switching costs without representing task rules or task sets. However, associative approach between cue, target, and response as suggested by Forrest et al. (2014) is not compatible with the associative structure in the present experiments. In our experiments, each target had additional unique features and participants could select the correct response by association irrespective of task cues. Performance as a result of associative learning should, therefore, resemble performance in studies employing univalent targets (Dreisbach et al., 2006, 2007; Dreisbach and Haider, 2008). We also predicted that participants who do not apply task rules and perform by association should not exhibit task-switching costs (see also Li et al., 2017).

## Experiment 1

In Experiment 1 we manipulated the use of task cues and task rules in three successive stages. We instructed participants to memorize target-response associations in Stage 1. In Stage 2 we displayed task cues without task rules. In Stage 3, we displayed task cues and participants were instructed to apply task rules as in a typical task-cueing paradigm (Meiran, 2014). The reason for displaying the task cues in each trial of Stage 2 was to introduce cue-related distraction. This should make performance in Stage 2 and 3 more comparable.

We made the following predictions based on the theoretical accounts outlined above. According to mediated response selection (Schneider, 2015, 2017) participants should show significant response-congruency effects in Stage 3, because they were able to apply the task rules while target feature-categorizations according to the task rules activate conflicting responses in incongruent trials.

In Stage 1 and 2, participants were unable to apply the task rules. However, participants may passively learn the bivalent task-relevant features of the targets without invoking rule-based processing (Hommel, 1998, 2004). If these bivalent features are associated with responses then they may lead to conflicting responses in incongruent trials. We therefore predicted that participant would also show significant response-congruency effects in Stage 1 and 2.

Note that since each target also had a task-irrelevant feature associated with a single response, if participants learn to associate this task-irrelevant target feature with a response, there should be no conflicting target-response associations. The response selection via the non-mediated route should not produce any congruency effects in all stages.

Similar to previous results (c.f., Kiesel et al., 2010; Vandierendonck et al., 2010) participants should show significant task-switching costs in Stage 3 where participants were instructed about the task rules. In Stage 1 and 2, according to results in previous task-switching studies on associative learning using univalent targets (Dreisbach et al., 2006, 2007; Dreisbach and Haider, 2008), participants should show no task-switching costs because participants will associate the task-irrelevant features with the responses but do not know the task rules.

### Methods

#### Participants

Twenty-four (18 females) university students from the University of Glasgow participated in this experiment (*M* = 22 years, *SD* = 3.8). Each student received £3 for their participation. The study was carried out in accordance with the recommendations of the BPS Code of Ethics and Glasgow University College of Science and Engineering Ethics Committee. All participants gave written consent to take part.

#### Apparatus and Stimuli

The experiment was programmed using PsyToolkit (Stoet, 2010, 2017). All stimuli were presented centrally on a 24-inch BenQ computer monitor. A Black Box Toolkit response pad was used to record participants’ responses with ± 1 ms resolution. Participants gave left and right responses by pressing a corresponding button with their left and right index finger, respectively. There were eight bivalent target stimuli, and each target stimulus had bivalent features color (black or white) and shape (circle or hexagon) and at least one additional task-irrelevant feature. These additional task-irrelevant features made each target stimulus unique (see Figure 1) suggesting a single associated response key. The two task cues were different surrounds: A multi-colored outline of an octagon frame served as the color task cue and a black square served as the shape task cue. The size of each target stimulus was 17 × 17 mm, and the size of each task cue was 34 × 34 mm. All stimuli were presented on a dark green background (RGB: 128, 150, 0).

#### Procedure

Participants first signed the consent form before they were seated in front of the computer screen at a viewing distance of approximately 50 cm. They read the on-screen instructions before they completed the three stages of the experiment. More specific instructions were displayed on screen before each stage.

In Stage 1, the participants were informed about the eight target stimuli (see Figure 1). The instructions stated that the four targets on the left hand side of the screen should result in a left-key response, and the four targets on the right hand side should result in a right-key response. Participants were asked to remember the targets and their corresponding keys. In each trial one of the eight possible target stimuli was displayed. No task cues were presented. Since each target stimulus triggered only a single target-response association, the participants should be able to recall the correct response without the help of task cues. Participants first carried out a training block with 16 trials followed by an experimental block with 64 trials in Stage 1.

In each trial of Stage 2 and 3 the target stimulus, and the task cue were presented simultaneously, with the target displayed inside a surround that served as a cue. In Stage 2, participants were instructed that the surround was meaningless and should be ignored when responding to the targets. Participants completed two experimental blocks with 100 trials each.

In Stage 3, each participant was informed about the task cues and related task rules. The instructions stipulated two tasks and task rules. For the color task, participants had to determine if the color of a target stimulus was mainly black or mainly white (white ⇒ press the left key; black ⇒ press the right key). For the shape task, participants had to determine if the shape of a target stimulus was mainly a circle or mainly a hexagon (circle ⇒ press the left key; hexagon ⇒ press the right key; Figure 1).

As a consequence, participants could use the task rules or target-response associations to give a response. All participants completed two experimental blocks with 100 trials each. In each trial, if a correct response was made, the next trial would commence after a 300 ms inter-trial interval. If no response was made within 2.5 seconds, the text message “Timeout” was displayed. Incorrect responses were followed by the on-screen text message “Mistake”. Both feedbacks were visible for 3 seconds before the next trial started.

#### Data Analyses

In the following, error trials were excluded from RT analyses. The first trial of each block and trials immediately following an incorrect response were excluded from all analyses. If participants made an error in *trial n - 1*, the subsequent *trial n* cannot be classified as a switch or repeat trial. Moreover, if *trial n - 1* and *trial n* had the same cue-target combination, then this identical-repeat *trial n*, was also removed from all analyses because participants could simply repeat the same response without engaging in the task. We also excluded all training trials from the analyses. In total, 3.9, 8.8, and 8.6% of the data were removed from the experimental block(s) in Stage 1, Stage 2 and Stage 3, respectively. For all data analyses, we used statistical software package R, version 3.4.2 (R Core Team, 2017). Raw data are available in Supplementary Materials.

### Results

**FIGURE 2:**
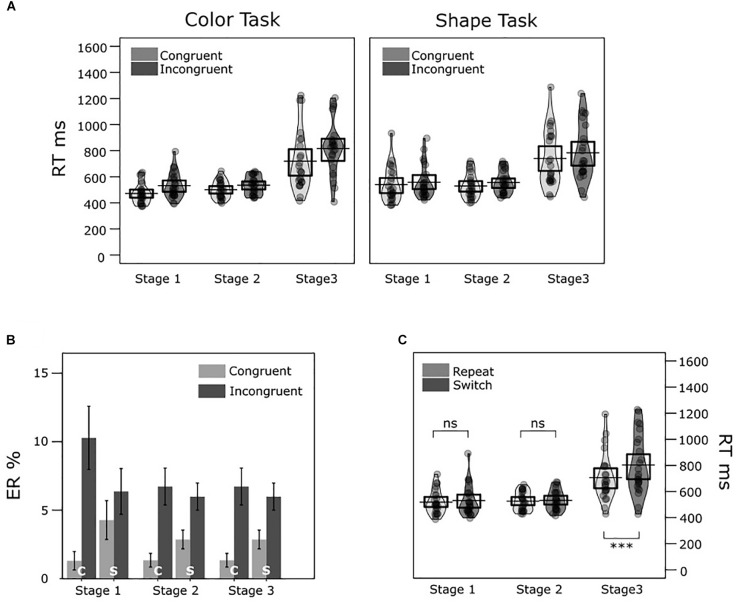
Experiment 1 mean response times (RTs) and error rates (ERs) across conditions. **(A)** The two violin plots illustrate the RT distributions in congruent and incongruent conditions across stages in the color task and shape task, respectively. The jittered dots inside each bean represent averaged RTs of each participant. The black horizontal bar and the box around it represent the mean and the 95% CI of the mean in each condition, respectively. **(B)** Bar charts show mean ERs in each condition (c = color/ s = shape task with congruent/ incongruent targets in Stage 1, 2, and 3). The error bars denote ± 1 *SEM*. **(C)** Similar violin plots illustrate RT distributions for the repeat and switch conditions across stages. ns = non-significant, ^∗∗∗^*p* < 0.001.

Two four-way ANOVAs with repeated measurements were conducted on mean RTs and ERs to compare performance in different conditions. The four factors were Task (color, shape), Trial transition (switch, repeat), Congruency (congruent, incongruent) and Stage (Stage 1, Stage 2, and Stage 3). We entered the factor “Task” to control for asymmetries in task difficulty. Including this factor can provide a better understanding of response-congruency effects in different conditions (e.g., Schneider, 2017). The results of the analyses are summarized in Table 1 and illustrated in Figure 2. Mean results of each condition are listed in the Appendix.

**Table 1:** Experiment 1: Results of ANOVA on RT and ER, with factors Task (color and shape), Trial transition (repeat and switch), Congruency (congruent and incongruent) and Stage (Stage 1, Stage 2, and Stage 3).

Factor	Response time				Error rate			
	*F*	*df*	*p*	${\text{η}}_{\text{p}}^{2}$	*F*	*df*	*p*	${\text{η}}_{\text{p}}^{2}$
Task	4.00	1,23	0.058	0.148	0.37	1,23	0.549	0.016
Trial	29.05	1,23	<0.001	0.558	28.00	1,23	<0.001	0.558
C	47.26	1,23	<0.001	0.673	20.41	1,23	<0.001	0.470
S	45.40	2,46	<0.001	0.664	2.29	2,46	0.112	0.091
Task × Trial	0.56	1,23	0.463	0.024	0.08	1,23	0.777	0.004
Task × C	6.55	1,23	0.018	0.222	5.87	1,23	0.023	0.203
Trial × C	1.57	1,23	0.222	0.064	2.39	1,23	0.136	0.094
Task × S	4.17	2,46	0.022	0.153	1.24	2,46	0.300	0.051
Trial × S	20.80	2,46	<0.001	0.475	1.38	2,46	0.261	0.057
C × S	7.13	2,46	0.002	0.237	0.45	2,46	0.641	0.019
Task × Trial × C	0.82	1,23	0.374	0.034	0.18	1,23	0.671	0.008
Task × Trial × S	0.40	2,46	0.674	0.017	3.33	2,46	0.045	0.127
Task × C × S	2.19	2,46	0.124	0.087	1.52	2,46	0.230	0.062
Trial × C × S	1.56	2,46	0.221	0.064	0.35	2,46	0.708	0.015
Task × Trial × C × S	0.57	2,46	0.571	0.024	1.12	2,46	0.335	0.046

*Trial = Trial transition; C = Congruency; S = Stage.*

We found significantly longer mean RTs and higher mean ERs in task-switch trials (625 ms, 5.95%) compared to task-repeat trials (584 ms, 3.27%). We also found significantly slower responses and more errors in incongruent trials (628 ms, 6.94%) than in congruent trials (581 ms, 2.28%). In the following *post hoc* pairwise comparisons were always adjusted for multiple comparisons after Holm (1979). When comparing RT differences between stages, the RT difference between Stage 1 (mean = 524 ms) and Stage 2 (mean = 530 ms) was not significant, *t*(23) = -0.66, *p* = 0.518. The difference between Stage 1 and Stage 3 (mean = 759 ms) was statistically significant, *t*(23) = -7.03, *p* < 0.001; as well as the difference between Stage 2 and Stage 3, *t*(23) = -6.71, *p* < 0.001.

The interaction between task and congruency (Task × C) was significant in both the RT and ER analyses. For RT, congruency effects were statistically significant in the color task (incongruent – congruent = 64 ms), *t*(23) = 6.38, *p* < 0.001; and shape task (30 ms), *t*(23) = 3.23, *p* < 0.001). A *post hoc* comparison indicated larger congruency effects in the color task than in the shape task, *t*(23) = 2.56, *p* = 0.018. For ER, congruency effects were also significant in both color task (incongruent – congruent = 6.86%), *t*(23) = 3.84, *p* < 0.001; and shape task (2.46%), *t*(23) = 3.24, *p* = 0.004. A *post hoc* comparison indicated that congruency effects were larger in the color task than in the shape task, *t*(23) = 2.42, *p* = 0.024.

Task significantly interacted with Stage (Task × S) in the RT analysis. The task differences were significant in Stage 1 (shape - color = +45 ms), *t*(23) = 2.98, *p* = 0.007); and Stage 2 (+25 ms), *t*(23) = 2.82, *p* < 0.010); but not in Stage 3 (-5 ms), *t*(23) = -0.24, *p* = 0.809.

The interaction between trial transition and stage (Trial × S) was also statistically significant in the RT analysis. The *post hoc* pairwise comparisons suggested RT switch costs were statistically significant in Stage 3 (switch – repeat = 100 ms), *t*(23) = 5.53, *p* < 0.001; but not in Stage 1 (15 ms), *t*(23) = 1.70, *p* = 0.102; and Stage 2 (5 ms), *t*(23) = 1.24, *p* = 0.226. For ER, *post hoc* comparisons showed that ER switch costs were significant in Stage 1 (switch – repeat = 3.76%), *t*(23) = 3.21, *p* = 0.025; and Stage 3 (2.45%), *t*(23) = 3.74, *p* = 0.009; but not in Stage 2 (1.83%), *t*(23) = 1.80, *p* = 0.094.

We also observed a significant interaction between Congruency and Stage (C × S) in the RT analysis. The *post hoc* pairwise comparisons suggested RT congruency effects were statistically significant in three stages, with *p* < 0.001: Stage 1 had a congruency effect of incongruent - congruent = 39 ms, *t*(23) = 5.78; Stage 2 had a congruency effect of 30 ms, *t*(23) = 5.85; and Stage 3 had a congruency effect of 71 ms, *t*(23) = 5.06. The congruency effects were equivalent in Stage 1 and Stage 2, *t*(23) = 1.40, *p* = 0.174. However, the congruency effects were larger in Stage 3 than in Stage 1, *t*(23) = 2.39, *p* = 0.026; and larger in Stage 3 than in Stage 2, *t*(23) = 3.15, *p* = 0.005. A *post hoc* comparison indicated that ER congruency effects were significant for all stages, with *p* < 0.05, but not significant across stages, with *p* > 0.05. Task significantly interacted with trial transition and stage (Task × Trial × S) in the ER analysis. However, this interaction was not predicted.

#### Verbal Report

**FIGURE 3:**
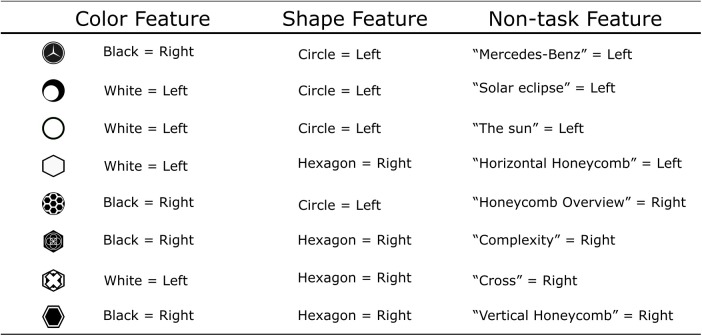
Features and responses in Experiment 1. Each stimulus had two task-relevant features (Color and Shape) and at least one non-task or task-irrelevant feature. Participants can make a correct response by employing a non-task feature directly. The non-task features listed in the right are reported by participants after the experiment (i.e., “Mercedes-Benz”, “Solar Eclipse”). Examples are listed for illustrative purposes only as each participant labeled the non-task feature differently.

All participants reported that they applied the task rules in Stage 3. In contrast, participants reported that they applied target-response associations in Stages 1 and 2. Since each target stimulus had a unique task-irrelevant feature, participants reported that they directly associated these task-irrelevant or non-task feature with the response (for example, “Mercedes-Benz” logo = > left key; see Figure 3).

### Discussion

In Experiment 1, participants were instructed to apply target-response associations in Stages 1 and 2 and task rules in Stage 3. In line with our predictions, we observed significant RT and ER response-congruency effects in all three stages. The response-congruency effects in Stage 3 were not surprising because participants, who were instructed to apply the task rules, categorized the bivalent target features. According to the mediated route account (Meiran and Kessler, 2008; Schneider and Logan, 2009, 2014; Reisenauer and Dreisbach, 2014; Schneider, 2015, 2017), target-feature categorization with respect to two tasks should lead to response-congruency effects.

However, we also found significant response-congruency effects in Stage 1 and 2. Since each target had a unique task-irrelevant feature associated with a single response, there should be no conflict between target-response associations in the non-mediated route of response selection. Indeed, participants reported that they associated the task-irrelevant features with responses in Stage 1 and 2 (Figure 3). In other words, participants gave correct responses without actively categorizing features of the target according to the task rules. As suggested by Hommel (1998, 2004), it seems likely that associations between the bivalent features and responses were passively formed, introducing response-congruency effects even though participants did not apply the task rules.

The RT task-switching results of Experiment 1 matched our prediction. Stage 1 had a non-significant switch cost of 15 ms and Stage 2 had a non-significant switch cost of 5 ms. In contrast, the task-switching costs were significant in Stage 3 amounting to 100 ms. Forrest et al. (2014) employed bivalent targets and found significant switch costs when participants did not apply task rules. A critical difference between their study and our Experiment 1 was the design of the targets. In our study each target had unique task-irrelevant features so that participants could directly link the unique features with a response, similar to the univalent targets in other studies (Dreisbach et al., 2006, 2007; Dreisbach and Haider, 2008). The ER results of Experiment 1 matched our prediction only partially because ER task-switching costs were also significant in Stage 1. However, in Stage 1 no task cues were presented and no task rules were introduced, and therefore participants could not switch between color and shape tasks. Since Stage 1 had only 64 trials with relatively low ERs (< 10%), mean ERs are the result of relatively few observations in each condition. In order to estimate ER switch costs more reliably participants had to complete 200 trials in Stage 1 of Experiment 2.

## Experiment 2

In Experiment 2, we sought to replicate the results of Experiment 1 by showing that the response-congruency effect would remain statistically significant regardless of the application of task rules. Since we had in Experiment 1 eight different target stimuli, four congruent and four incongruent targets, it is possible that the response-congruency effect was related to the specific design of our targets. For example, the four incongruent target stimuli employed in Experiment 1 may have been more difficult to recall than the four congruent target stimuli. This is possible because target stimuli had different unique features (Figure 1 and Figure 3).

**FIGURE 4:**
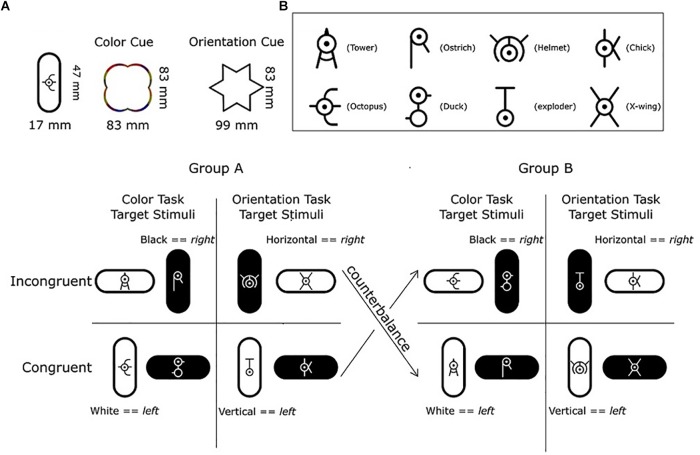
Illustration of the stimuli in Experiment 2. **(A)** Each target stimulus consisted of a vertically or horizontally oriented lozenge shape in Black or White with one of eight different symbols inside. The unfamiliar symbol for the congruent/incongruent target stimuli were counterbalanced between two groups of participants (Group A, Group B). Within each group, four symbols were only presented in the color task, and the other four symbols were only presented in the shape task. **(B)** List of the eight unfamiliar symbols. Labels for the symbols such as “Tower”, “Helmet” and “Chick” were compiled from post-experiment verbal reports and are shown in parentheses. They are for illustrative purposes only as participants may have used a different label.

Here, we designed new targets in order to avoid any target-specific effect on response-congruency. In Experiment 2, we assigned an unfamiliar symbol to each target stimulus. These task-irrelevant symbols were derived from letters in the Greek alphabet (Figure 4). We counterbalanced the combination of symbols and congruent/incongruent targets between two groups of participants to eliminate any target-specific effect (Figure 4).

### Methods

#### Participants

A new sample of twenty-four (17 female) university students from the University of Glasgow participated in this experiment (*M* = 24 years, *SD* = 2.9). Each student received £3 for their participation.

#### Apparatus and Stimuli

All stimuli were presented on a 24-inch BenQ computer monitor. A Black Box Toolkit response pad was used to record responses from each participant. The size of all target stimuli was 47 × 17 mm (vertical lozenge shape) or 17 × 47 mm (horizontal lozenge shape). The target stimuli were vertical or horizontal and black or white. A multi-colored outline of a cloud served as the cue for the color task (83 × 83 mm) and a black outline of a star (hexagram) served as the cue for the orientation task (99 × 83 mm; Figure 4). All stimuli were presented on a dark green background (RGB: 128, 150, 0). Two sets of eight target stimuli were created and assigned to two groups of participants (Group A and B). Each set of target stimuli was created by all combinations of color (Black, White) and orientation (Vertical, Horizontal) and by inserting eight unfamiliar symbols inside the shape. The different symbols and their use in congruent/incongruent target stimuli were counterbalanced between Group A and B. Within each group, each task was assigned to four corresponding target stimuli (see Figure 4).

#### Procedure

The procedure was almost identical as in Experiment 1 with only two exceptions. First, in order to counterbalance the potential target-specific effect, each participant was randomly assigned to one of the two target-stimulus groups (Figure 4). Second, in Stage 1 of Experiment 2, participants first carried out a 20-trial training block followed by two 100-trial experimental blocks with the two tasks randomly intermixed. In Experiment 2, task rules stipulated the color task and orientation task. For the color task, participants had to determine if the background of a target was white or black (white ⇒ press the left key; black ⇒ press the right key). For the orientation task, participants had to determine if the orientation of a target was vertical or horizontal (vertical ⇒ press the left key; horizontal ⇒ press the right key).

#### Data Analyses

Similar exclusion criteria as in Experiment 1 were used, except that no identical-repeat trials had to be removed because we controlled the cue-target combinations so that the same combination did not occur in consecutive trials. As a result, only 1.1, 1.1, and 1.3% of the data had to be removed from the experimental blocks in Stage 1, Stage 2 and Stage 3, respectively.

### Results

**FIGURE 5:**
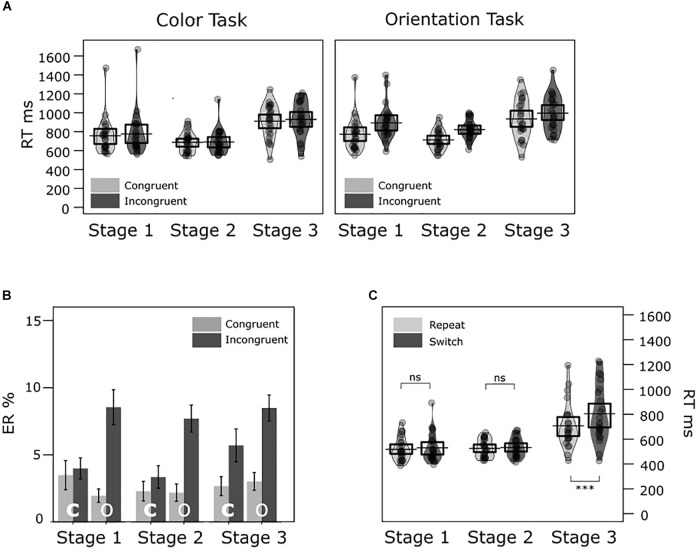
Experiment 2 mean RTs and ERs across conditions. **(A)** The two violin plots illustrate the RT distributions in congruent and incongruent conditions across stages in the color task and orientation task, respectively. The jittered dots inside each bean represent averaged RTs of each participant. The black horizontal bar and the box around it represent the mean and the 95% CI of the mean in each condition, respectively. **(B)** Bar charts show mean ERs in each condition (c = color/ o = orientation task with congruent/ incongruent targets in Stage 1, 2, and 3). The error bars denote ± 1 *SEM*. **(C)** Similar violin plots illustrate RT distributions for the repeat and switch conditions across stages. ns = non-significant,^∗∗∗^*p* < 0.001.

Two four-way ANOVAs with repeated measurements were conducted on mean RTs and ERs to compare different conditions. The between-subjects factor Group was dropped because counterbalancing of targets across participants had no statistically significant effects on RT and ER. The four within-subjects factors were Task (color, orientation), Trial transition (repeat, switch), Congruency (congruent, incongruent) and Stage (Stage 1, Stage 2, and Stage 3). The results of both analyses are summarized in Table 2 and illustrated in Figure 5. Mean values from each condition are listed in the Appendix.

**Table 2:** Experiment 2: Results of ANOVAs on mean RTs and ERs, with Task (color, orientation), Trial transition (repeat, switch), Congruency (congruent, incongruent), and Stage (Stage 1, Stage 2, Stage 3) as within-subjects factors.

Factor	Response time				Error rate			
	*F*	*df*	*p*	${\text{η}}_{\text{p}}^{2}$	*F*	*df*	*p*	${\text{η}}_{\text{p}}^{2}$
Task	36.57	1, 23	<0.001	0.614	6.40	1, 23	0.019	0.218
Trial	40.49	1, 23	<0.001	0.638	6.99	1, 23	0.014	0.233
C	26.22	1, 23	<0.001	0.533	32.85	1, 23	<0.001	0.588
S	26.46	2, 46	<0.001	0.535	1.41	2, 46	0.255	0.058
Task × Trial	0.17	1, 23	0.684	0.007	0.37	1, 23	0.547	0.016
Task × C	16.04	1, 23	<0.001	0.411	15.19	1, 23	<0.001	0.398
Trial × C	1.40	1, 23	0.249	0.057	1.70	1, 23	0.205	0.069
Task × S	0.62	2, 46	0.544	0.026	0.10	2, 46	0.904	0.004
Trial × S	49.91	2, 46	<0.001	0.685	1.05	2, 46	0.359	0.044
C × S	1.16	2, 46	0.322	0.048	0.31	2, 46	0.737	0.013
Task × Trial × C	0.72	1, 23	0.405	0.030	3.63	1, 23	0.069	0.136
Task × Trial × S	1.78	2, 46	0.180	0.072	3.19	2, 46	0.051	0.122
Task × C × S	2.21	2, 46	0.121	0.088	0.87	2, 46	0.426	0.036
Trial × C × S	1.93	2, 46	0.156	0.078	0.56	2, 46	0.573	0.024
Task × Trial × C × S	1.58	2, 46	0.217	0.064	0.13	2, 46	0.880	0.006

*Trial, Trial transition; C, Congruency; S, Stage.*

Response times were longer and ERs were higher in the orientation task (856 ms, 5.32%) compared to the color task (793 ms, 3.58%), in switch trials (856 ms, 4.96%) compared to repeat trials (793 ms, 3.94%), and in incongruent trials (852 ms, 6.30%) compared to congruent trials (797 ms, 2.60%). For the factor of Stage in the RT analysis, *post hoc* pairwise comparisons, corrected after Holm (Holm, 1979), were conducted to test differences in mean RT between the three stages. The results showed that RT difference between Stage 1 (800 ms) and Stage 2 (729 ms) was statistically significant, *t*(23) = 3.27, *p* < 0.003. The difference between Stage 1 and Stage 3 (944 ms) was significant, *t*(23) = -4.03, *p* < 0.001; and the difference between Stage 2 and 3 was significant, *t*(23) = -6.90, *p* < 0.001.

Task significantly interacted with congruency (Task × C) in both RT and ER analyses. For RT, the congruency effects were statistically significant in the orientation task (incongruent - congruent = 95 ms), *t*(23) = 5.28, *p* < 0.001; but not in the color task (15 ms), *t*(23) = 1.46, *p* = 0.157. For ER, the congruency effects were significant in both orientation task (5.86%), *t*(23) = 6.95, *p* < 0.001; and color task (1.53%), *t*(23) = 1.78, *p* = 0.046. A *post hoc* comparison showed larger congruency effects in the orientation task than in the color task, *t*(23) = 3.90, *p* < 0.001.

We also found a significant interaction between Trial transition and Stage (Trial × S) in the RT analysis. A *post hoc* comparison indicated that the RT switch costs were not significant in Stage 1 (switch - repeat = 4 ms), *t*(23) = 0.681, *p* = 0.503; and Stage 2 (8 ms), *t*(23) = 1.77, *p* = 0.091; but statistically significant in Stage 3 (177 ms), *t*(23) = 7.07, *p* < 0.001. A *post hoc* comparison on ER switch costs across stages indicated no statistically significant differences across stages.

RT congruency effects were significant in all three stages, with *p* < 0.001: In Stage 1 the RT congruency effect was 68 ms, *t*(23) = 3.26; in Stage 2 it was 56 ms, *t*(23) = 4.45; and in Stage 3 the RT congruency effect was 42 ms, *t*(23) = 5.46. ER congruency effects were also statistically significant in all three stages, with *p* < 0.001: In Stage 1 the ER congruency effect was 3.55%, *t*(23) = 3.54; in Stage 2 it was 3.28%, *t*(23) = 3.85; and in Stage 3 the ER congruency effect was 4.26%, *t*(23) = 3.96. RT and ER congruency effects were not statistically different across the three stages.

#### Verbal Report

All participants reported that they applied the task rules in Stage 3. In contrast, they reported that they applied target-response associations in Stage 1 and 2 as in Experiment 1. Since each stimulus had a unique symbol with task-irrelevant target features, the participants reported that they had linked these symbols directly to the response key (for example, Octopus ⇒ press the left key; see Figure 4).

### Discussion

As predicted and in line with the results of Experiment 1, participants showed RT and ER congruency effects in all three stages. Since we carefully controlled any target-specific effects in Experiment 2, we propose that response-congruency effects were the result of conflicts between bivalent feature-response associations in the incongruent trials.

Consistent with the results of Experiment 1 and previous studies (Dreisbach et al., 2006, 2007; Dreisbach and Haider, 2008), participants showed negligible RT and ER task-switching costs in Stage 1 and 2 when they used target-response associations. RT task-switching costs emerged in Stage 3 when participants applied task rules.

## General Discussion

In two experiments, we investigated whether participants show response-congruency effects when responding to target stimuli by association rather than applying task rules. We assigned an additional task-irrelevant feature to each bivalent target stimulus, so that participants could avoid response conflicts by directly associating the task-irrelevant target feature with a single response via the non-mediated route. Although this form of associative learning should not produce any response-congruency effects, we observed that participants showed response-congruency effects but no task-switching costs.

### Response-Congruence Effects

Previous studies have suggested that the mediated route plays an important role in response selection because response-congruency effects were observed when participants followed task rules and responded to target stimuli that were never repeated (Liefooghe et al., 2012; Schneider, 2015, 2017). In Stage 1 and 2 of our experiments participants were instructed to respond by association and we observed significant response-congruency effects. Participants could not apply task rules in Stage 1, simply because no task cues were presented. In their verbal reports participants also stated that they did associate the task-irrelevant target features with the corresponding responses in Stage 1 and 2. In other words, they ignored the bivalent target features (color, shape/orientation) during response selection. According to the proponents of the non-mediated route of response selection (Kiesel et al., 2007; Wendt and Kiesel, 2008; Waszak et al., 2013; Schneider and Logan, 2015), participants should not produce response-congruency effects.

The significant congruency effects observed in Stages 1 and 2 can be explained by passive associative learning (Hommel, 1998, 2004). Hommel proposed that features of target stimuli may be learned passively and automatically leading to conflicts during response selection even though the task rules are not applied explicitly. We suggest that, although participants performed the task by using task-irrelevant target-response associations, task-relevant feature-response associations were also formed because the same bivalent target stimuli were repeated throughout each experiment. These associative response mappings may be responsible for the significant response-congruency effects in the first two stages of both experiments.

Passive learning and response mapping are also characteristics of the well-known flanker effect. In this paradigm a target stimulus is presented at the center of a screen which is flanked by either response-congruent or response-incongruent distractors. Researchers observed that RTs were shorter and accuracy better for congruent flankers than for incongruent flankers, irrespective of participants applying rule-based instructions, using stimulus-response associations that have already been practiced or using newly instructed stimulus-response associations (e.g., Eriksen and Eriksen, 1974; Wenke et al., 2014). For example, when using newly instructed stimulus-response mappings, participants showed a significant flanker-congruency effect in their RTs, even at the very beginning of the block (Wenke et al., 2014).

### Response-Congruence Effects and Task Rules

Applying task rules can invoke conflicts between feature-response associations and produce response-congruency effects even when targets are never repeated (Liefooghe et al., 2012; Schneider, 2015, 2017). Our results suggest that in paradigms with repeated targets response-congruency effects do not need to be the result of rule-based feature categorization, and instead that target-response associations may be formed passively and automatically (Hommel, 1998, 2004). This raises the question whether applying task rules in addition to passively learned bivalent target response associations can increase response-congruency effects.

In Experiment 1, participants showed increased response-congruency effects in Stage 3 when they applied task rules. In Experiment 2, however, the response-congruency effects were similar and did not vary across stages. In Experiment 1, each target had multiple task-irrelevant features, so that the bivalent target features (color and shape) were less salient and therefore less likely to be used for response retrieval in the first two stages. In Stage 3 of Experiment 1, however, the task rules explicitly referred to the task-relevant target features increasing the response-congruency effects. In Experiment 2, each target had a unique and unfamiliar symbol superimposed (such as “Tower”, “Helmet” and “Chick”), so that the bivalent target features (color and orientation) were consistent and salient across targets and trials. Thus, participants may have formed task-relevant feature-response associations in Stage 1 and 2 so that the introduction of the task-rules in Stage 3 no longer increased the response-congruency effects. We suggest that in paradigms with repeated target stimuli a sizable portion, if not all, of the response-congruency effects, may be attributed to passively learned feature-response mappings.

### Response-Congruence Effects in Different Tasks

Interestingly, we also found that task type modulated the response-congruency effects in both experiments. In Experiment 1, we did not counterbalance the combination of non-task relevant features and the congruent/incongruent targets. Thus, the interaction between task type and response congruency may be due to target-specific effects. For example, since each target had task-irrelevant features, the incongruent targets of the color task may be more difficult to remember than the incongruent targets of the shape task, resulting in larger response-congruency effects for the color task than for the shape task.

In Experiment 2, the results indicate that participants had stronger response-congruency effects in the orientation task than in the color task. Since we counterbalanced the combination of task-irrelevant features and the congruent/incongruent targets we can rule out a possible target-specific effect. The interaction between task and response-congruency effects may be explained by “categorization difficulty” (Meiran and Kessler, 2008; Schneider, 2017). Schneider (2017) suggested that response-congruency effects were larger when “categorization” of the irrelevant task dimensions was easier. Similarly, even when participants did not apply task rules, some features may be easier to detect than others. In the orientation task, for example, color features were irrelevant and might have been easier to detect because participants responded more quickly in the color task. If color is picked up more quickly than orientation, then this may produce stronger response-congruency effects in the orientation task than in the color task. Future studies may manipulate the difficulty of the two tasks in order to determine whether this modulates response-congruency effects when using associative learning.

### Task-Switching Costs

Previous studies have shown that target-response associations can only eliminate task-switching costs when the target stimuli were univalent (Dreisbach et al., 2006, 2007; Dreisbach and Haider, 2008). If the targets were bivalent, target-response associations did not eliminate task-switching costs (Forrest, 2012; Meier et al., 2013; Forrest et al., 2014). The results of Experiment 1 and 2 suggest that when participants establish unique target-response associations, the task-switching costs are non-significant for bivalent target stimuli. This resembles task-switching results for univalent targets (Dreisbach et al., 2006, 2007; Dreisbach and Haider, 2008). In addition, and in line with Dreisbach et al. (2006, 2007), and Dreisbach and Haider (2008), we found that participants responded more slowly and showed significantly increased switching costs when they applied task rules rather than associative learning. The switch costs are likely to reflect additional cognitive processing in task-switching trials, such as reconfiguring the task set and resolving proactive interference (e.g., Kiesel et al., 2010; Vandierendonck et al., 2010).

In Stage 1 and Stage 2, participants were instructed to use target-response associations while in Stage 3 they were instructed to apply the task rules. Although participants learned to associate responses with the targets in the first two stages, it seems that they preferred task rules in Stage 3 because they showed significant switch costs in Stage 3. Given that in everyday settings tasks might involve variable target stimuli, applying a rule-based strategy by default, may be more efficient when switching between tasks. We conclude that human participants tend to develop and prefer strategies that prioritize goal-relevant information in order to reduce uncertainty and cognitive effort (Mackie et al., 2013; Cooper et al., 2015).

### Future Research

The post-experiment verbal reports suggested that in Stage 1 and 2, all participants followed the instructions and none of the participants employed an alternative strategy, e.g., task-rule based strategy. Indeed, participants as a group did not demonstrate any task-switching costs suggesting that task rules were not applied. However, it is difficult to control or monitor participants’ strategies or rules during an experiment (Li et al., 2019). In addition, the post-experimental self-reports may not be entirely reliable because participants may have learned the task rules implicitly. One way of testing if participants implicitly learned the task rules would be to occasionally introduce a new target stimulus that did not appear before but has the relevant task features that are compatible with the task rules. Only participants who learned the task rules implicitly should be able to perform above chance.

## Conclusion

Consistent with previous studies on task-switching we showed in two experiments that participants responded more slowly and had significant task-switching costs when task rules were introduced. However, because we added unique features to the bivalent targets participants could also learn to respond by using target-response associations without applying the task rules. As a result of this associative learning participants were able to respond more quickly and to eliminate switch costs in Stages 1 and 2. In addition, we found significant congruency effects across all stages, suggesting that although task rules were not applied, bivalent task features may be learned passively, resulting in response-selection conflicts for incongruent targets. When participants apply task rules in experiments where target stimuli are repeated, we suggest that the mediated route of response selection may play a less prominent role in producing response-congruency effects than previously assumed.

## Author Contributions

All authors listed have made a substantial, direct and intellectual contribution to the work, and approved it for publication.

## Conflict of Interest Statement

The authors declare that the research was conducted in the absence of any commercial or financial relationships that could be construed as a potential conflict of interest.

## Supplementary Material

DATA SHEET S1 Dataset in Experiment 1 and 2.

## APPENDIX

**Table A1:** Experiment 1: Mean (SE) of RT and ER for each trial condition and stage.

Task/Trial/Stage	Stage 1		Stage 2		Stage 3	
*Color*	RT ms (*SE*)	ER % (*SE*)	RT ms (*SE*)	ER % (*SE*)	RT ms (*SE*)	ER % (*SE*)
Repeat Con	470 (14.56)	0.00 (0)	496 (12.51)	0.37 (0.26)	667 (44.43)	1.15 (0.42)
Repeat Inc	519 (20.22)	9.95 (3.22)	530 (15.67)	5.03 (1.68)	749 (39.51)	5.33 (1.25)
Switch Con	475 (18.30)	2.62 (1.31)	505 (14.74)	2.32 (0.93)	756 (50.19)	2.45 (0.65)
Switch Inc	542 (20.71)	10.61 (3.38)	541 (14.39)	8.43 (2.10)	872 (43.43)	10.73 (2.31)
*Shape*						
Repeat Con	522 (24.18)	2.27 (1.07)	530 (18.46)	2.61 (0.81)	695 (44.03)	1.59 (0.63)
Repeat Inc	555 (24.00)	2.50 (1.55)	555 (19.40)	5.26 (1.55)	725 (35.57)	3.13 (0.93)
Switch Con	552 (33.35)	6.28 (2.59)	530 (20.49)	3.11 (1.11)	775 (46.89)	2.51 (0.93)
Switch Inc	558 (28.49)	10.24 (2.79)	556 (16.79)	6.72 (1.24)	831 (52.77)	5.32 (1.45)
Repeat	516 (10.84)	3.68 (0.99)	528 (8.51)	3.32 (0.63)	709 (20.45)	2.80 (0.46)
Switch	532 (13.20)	7.44 (1.33)	532 (8.47)	5.14 (0.74)	809 (24.30)	5.25 (0.81)
Congruent	505 (12.16)	2.79 (0.80)	515 (8.43)	2.10 (0.43)	723 (23.30)	1.93 (0.34)
Incongruent	544 (11.71)	8.33 (1.43)	545 (8.28)	6.36 (0.83)	794 (22.16)	6.13 (0.82)
Total	524 (8.54)	5.56 (0.84)	530 (5.99)	4.23 (0.49)	759 (16.24)	4.03 (0.47)

*Con, Congruent; Inc, Incongruent.*

**Table A2:** Experiment 2: Mean (SE) of RT and ER for each trial condition and stage.

Task/Trial/Stage	Stage 1		Stage 2		Stage 3	
*Color*	RT ms (*SE*)	ER % (*SE*)	RT ms (*SE*)	ER % (*SE*)	RT ms (*SE*)	ER % (*SE*)
Repeat Con	751 (41.09)	2.89 (1.01)	685 (23.12)	1.66 (0.68)	822 (27.45)	1.56 (0.59)
Repeat Inc	783 (49.59)	3.56 (0.96)	673 (25.64)	2.55 (0.76)	848 (32.87)	5.44 (1.80)
Switch Con	764 (38.96)	4.08 (1.95)	692 (20.97)	2.93 (1.29)	1000 (47.34)	3.78 (1.26)
Switch Inc	769 (45.08)	4.42 (1.26)	712 (28.47)	4.14 (1.50)	1020 (47.81)	5.97 (1.68)
*Orientation*						
Repeat Con	782 (32.98)	1.61 (0.57)	722 (21.53)	3.17 (1.19)	844 (37.21)	3.54 (1.06)
Repeat Inc	876 (36.66)	6.16 (1.30)	818 (21.91)	7.19 (1.12)	909 (35.89)	7.93 (1.26)
Switch Con	768 (39.87)	2.31 (0.82)	702 (23.51)	1.20 (0.46)	1028 (52.87)	2.49 (0.80)
Switch Inc	909 (37.92)	10.94 (2.17)	823 (20.71)	8.21 (1.69)	1083 (47.17)	9.05 (1.47)
Repeat	798 (20.52)	3.55 (0.52)	725 (12.79)	3.64 (0.52)	856 (16.85)	4.62 (0.66)
Switch	802 (20.93)	5.44 (0.87)	732 (12.83)	4.12 (0.70)	1033 (24.24)	5.32 (0.71)
Congruent	766 (18.90)	2.72 (0.60)	700 (11.07)	2.24 (0.48)	924 (22.86)	2.84 (0.48)
Incongruent	834 (21.86)	6.27 (0.79)	757 (13.76)	5.52 (0.69)	965 (22.48)	7.10 (0.79)
Total	800 (14.62)	4.50 (0.51)	729 (9.04)	3.88 (0.44)	944 (16.06)	4.97 (0.48)

*Con, Congruent; Inc, Incongruent.*
